# Public knowledge, attitudes and practices of vehicle submersion incidents: a pilot study

**DOI:** 10.1186/s40621-019-0192-0

**Published:** 2019-06-03

**Authors:** Gerren K. McDonald, Cheryl A. Moser, Gordon G. Giesbrecht

**Affiliations:** 1 Faculty of Kinesiology and Recreation Management, University of Manitoba, Winnipeg, Canada; 2 Gupta Faculty of Kinesiology and Applied Health, University of Winnipeg, 515 Portage Avenue, Winnipeg, Manitoba R3B2E9 Canada

**Keywords:** Sinking car, Vehicle in water, Self-rescue, Traffic accident, Public education

## Abstract

**Introduction:**

Vehicle submersions account for up to 10% of all drownings in high-income countries. Reports indicate that occupants may be conscious and functional, but possibly making incorrect decisions for self-rescue leading to drowning. This study investigated current public knowledge, attitudes and practices regarding vehicle submersion incidents and to determine if individuals, who are aware of educational efforts regarding vehicle submersions, indicated better responses.

**Method:**

A knowledge, attitude and practice (KAP) survey was developed based on previous findings and guidelines from *Operation ALIVE* (Automobile submersion: Lessons In Vehicle Escape) for vehicle submersion incidents.

**Results:**

The majority of respondents (87%) had knowledge of vehicle submersions from the media, but they were not aware (94%) of an effective self-rescue protocol. Respondents felt they had low risk of involvement in a vehicle submersion, and that the chance of survival was likely. Most respondents selected a “successful” initial action for escape; however, other responses indicate the chances of completing a successful self-rescue sequence was less likely. Only 45% of respondents were “aware” of *Operation ALIVE* educational initiatives, and this awareness did not generally produce better responses.

**Conclusions:**

Public understanding of vehicle submersion incidents is low and current public education efforts have not increased awareness in the severity or the urgency for performing self-rescue in this scenario. Simply increasing public knowledge of “SWOC” (“SEATBELTS” off, “WINDOWS” open, “OUT” immediately, “CHILDREN” first) would help to decrease the high fatality rate associated with this type of road traffic accident.

## Introduction

Road traffic accidents (RTA) have emerged as a major public health concern, and the United Nations launched the *Decade of Action for Road Safety (2011–2020)* (WHO 2011; Gopalakrishnan 2012). All types of RTAs need to be assessed in high-, middle- and low-income countries. Vehicle submersions have received little attention, yet they have one of the highest fatality rates of any type of single motor-vehicle accident, accounting for up to 10% of all drownings in high-income countries (e.g., Australia, Canada, Finland, New Zealand and USA) (McDonald and Giesbrecht 2013a).

Few vehicle submersion deaths result directly from traumatic injury, or indirectly from drowning due to trauma-induced incapacitation (Hammett 2007; SWOV 1973). Victims are usually conscious and potentially capable of performing self-rescue after their vehicle enters the water (Hammett 2007; Sternbrandt et al. 2008; Wintemute et al. 1990; Canadian Red Cross Society 2003). Occupants, who are conscious and functional after impact, still risk drowning due to either complete inactivity (panic/freezing), and/or incorrect actions (Giesbrecht 2005; Leach 1994) including: calling emergency dispatch; trying to open a door; letting the vehicle fill with water; relying on air bubble; or remaining in the vehicle because they feel safe, cannot swim, or are waiting for rescue (McDonald and Giesbrecht 2013a). It is likely that many of these drownings could be prevented if occupants knew what to do and acted quickly.

Clearly, preventing vehicle submersions would be the most effective strategy. Previous analyses have identified successful interventions that either: physically prevent vehicles from entering water [e.g., road design (road-curvature and lighting), guardrails and other barriers]; or provide warnings (e.g., signage, weather warning systems and speed reduction zones) (Hammett 2007; Sternbrandt et al. 2008; Wintemute et al. 1990; Maples and Tiefenbacher 2009; Staes et al. 1994).

Ultimately, much of the responsibility lies with drivers who must adjust speed and driving practices according to road and weather conditions (e.g., heavy traffic, road curvature, slippery surfaces and decreased visibility) to prevent entering water. Despite many prevention initiatives, and expectations of prudent driving practices, many vehicles still end up in water, due to drivers’ own actions or the actions of others (Hamilton et al. 2016). Thus, it is important that vehicle occupants not only know which practices are prudent, but also what to do in the unfortunate event their vehicle ends up in water.

In 2005 a research/educational program *Operation ALIVE* (Automobile submersion: Lessons In Vehicle Escape) responded to recommendations from an inquest into a vehicle submersion death (Howell 2005). Previous research and data on vehicle submersion accidents were reviewed (McDonald and Giesbrecht 2013b). A series of studies, involving more than 150 human-occupied vehicle, or simulator, submersions, was then conducted to answer several questions that had not been previously addressed (McDonald and Giesbrecht 2013a; Giesbrecht and McDonald 2010; Giesbrecht and McDonald 2011; Gagnon et al. 2012; Giesbrecht et al. 2017).

Key outcomes of the program included: a better understanding of vehicle sinking characteristics (e.g., determination of sinking phases and submersion times), the necessity for self-rescue and escape strategies; and the development of the “SWOC” acronym, a standardized best-practice escape protocol (“SEATBELTS” off, “WINDOWS” open, “OUT” immediately, “CHILDREN” first).

Public education efforts at this point have included: 1) provincial and state initiatives such as driver training handbooks (State of Indiana 2018; Manitoba Public Insurance 2018), public presentations/websites (Giesbrecht GG 2013), and televised public service announcements (Manitoba Public Insurance 2015); 2) national initiatives such as education and guidelines for industry (Winter Road Safety Committee M 2004; Winter Road Safety Committee 2004; Giesbrecht and Wilkerson 2006; Giesbrecht and Rankine 2007); and international initiatives such as changes to emergency dispatch response protocols (Giesbrecht 2016a; Giesbrecht 2016b).

The next steps/goals for the program are to evaluate current public knowledge on this topic and then generate, and advocate for, increased effective social media and online education materials. Currently, we are unaware of any evaluations of public knowledge related to vehicle submersions.

The aim of the present study was to conduct a public knowledge, attitudes and practices (KAP) survey related to vehicle submersion incidents. It was hypothesized that the majority of respondents would score poorly. A secondary aim was to identify if respondents who were “aware” of any *Operation ALIVE* initiatives, had improved responses compared to those who were “unaware”. It was hypothesized that “aware” respondents would score better than the “unaware” respondents. Results from this survey may help inform future initiatives to decrease vehicle submersion deaths, and direct further studies into public knowledge and practice.

## Methods

A public survey was conducted in a mid-sized Canadian city where many roadways run alongside or cross bodies of water. Respondents were a convenience sample of adults in the downtown area during a local cultural event. The University of Manitoba Education/Nursing Research Ethics Board approved the survey protocols. Respondents were eligible if they were 18 years old, provided written consent, and had not previously completed the survey.

### Survey tool development

A committee was used to develop a KAP survey based on previous findings and recommendations of the *Operation ALIVE* program (McDonald and Giesbrecht 2013a; Giesbrecht and McDonald 2010). A pilot version was administered to 15 respondents to evaluate interpretation, ambiguous wording and time to completion. Only minor edits were required. The final survey had 35 questions (28 closed-ended and 7 open-ended) and required 10–15 min to complete.

### Study instrument

The survey had four types of questions:

1. Demographics
  Four closed-ended questions regarding: age, sex, education level, and current vehicle used.
2. Knowledge
  Ten questions (6 closed-ended, 4 open-ended) regarding public education efforts (*n* = 2), awareness of vehicle submersion incidents (*n* = 3), knowledge of effective escape protocol (*n* = 2) and vehicle sinking characteristics (*n* = 3).
3. Attitudes and preferences
  Eight closed-ended questions regarding perceived risk of vehicle submersions (*n* = 2), chances of survival (*n* = 1), rescue tool colour and location (*n* = 3) and confidence in rescue tool use (*n* = 2).
4. Practice
  Thirteen questions (10 closed-ended, 3 open-ended) regarding the first escape action (*n* = 1), window breaking (*n* = 6), rescue tools (*n* = 2), and child seat use, location and restraint-type (*n* = 4).

For the final 5 questions, respondents sat in the driver’s seat of a demonstration car (Honda Civic) for completion of action-based responses, which were either visualized or performed. Six (yellow) rescue tools were pre-positioned in manufacture-recommended positions within the vehicle (keychain, driver side visor, center console, rear-view mirror, driver side door, and back of driver headrest).

### Data analysis

Data were analysed using SigmaPlot®. Fisher’s exact test, was first conducted to determine how the sample reflected the distribution of sex, age and education level of the Canadian population (Statistics Canada 2016a; Statistics Canada 2016b). All responses were summarized by frequency and, when appropriate, categorized as “correct” or “incorrect” based on evidence and guidelines from *Operation ALIVE* (McDonald and Giesbrecht 2013a*;* Giesbrecht and McDonald 2010*)*. If more than one response was provided for an open-ended question, the response was deemed “correct” if at least one of the responses was correct.

Responses from all respondents who were “aware” of educational efforts of *Operation ALIVE* were grouped and compared to those who were “unaware” of these efforts. Fisher’s exact test was also used to evaluate if these public education efforts positively affected responses. Significance for all analyses was set at *p* ≤ 0.05.

## Results

### Respondents

Survey respondents (*n* = 82) had a similar distribution for sex and age in comparison to the Canadian population, except for a smaller 75-y age group (Table 1). Compared to the Canadian population the level of education obtained was generally higher in the survey group. Seventy-one (87%) respondents reported currently using a vehicle; the types were sedan (43), SUV (13), truck (9), van (4) and coupe (2).

**Table 1:** Survey respondent demographics (*n* = 82). Canadian data for the highest level of education were only available for ages 25–64 (*n* = 66)

Characteristic	Sample n (%)	Population (%)	*p*-value
Sex			0.57
Female	45 (55)	(50)	
Male	37 (45)	(50)	
Age			
18-34 y	23 (28)	(29)	1.00
35-54 y	27 (33)	(34)	1.00
55-74 y	31 (38)	(29)	0.23
>75 y	1 (1)	(8)	0.03^*^
Highest education obtained			
High school diploma (or less)	8 (12)	(39)	0.0001^*^
University, diploma or certificate	18 (27)	(38)	0.13
Bachelor's degree (or higher)	40 (60)	(23)	0.0001^*^

_*_significant difference (*p* ≤ 0.05)

### Knowledge of public education efforts

Thirty-five (43%) respondents were “aware” of some public education through *Operation ALIVE* initiatives. They were “aware” through one or more of the following sources: television (33); and/or newspaper (7); radio (6); public presentations (2); or social media (1).

### Knowledge of incidents, escape protocol and sinking characteristics

Table 2 indicates that respondents, who were “aware” of previous educational initiatives, had more knowledge of media reports involving vehicle submersion, and the advised self-rescue protocol. No other significant effects were seen.

**Table 2:** Responses to “Knowledge” questions (*n* = 82; 5 closed-ended and 1 open-ended). Responses for the open-ended question were assigned as “correct” or “incorrect” time values

Questionnaire Item	Sample n (%)	Aware Group n (%)	Aware vs. Unaware *p*-value
Knowledge of media report(s) involving vehicle submersion			0.002^*^
Yes	71 (87)	35 (100)	
No	11 (13)	0 (0)	
Personal knowledge involving a vehicle submersion incident			0.50
Yes	10 (12)	3 (9)	
No	72 (88)	32 (81)	
Knowledge of the advised self-rescue protocol			0.01^*^
Yes	5 (6)	5 (14)	
No	77 (94)	30 (86)	
If a vehicle enters water intact and right-side-up, will it float right-side-up?			0.25
Yes (correct response)	63 (77)	25 (71)	
No (incorrect response)	19 (23)	10 (29)	
If a vehicle that enters the water intact and upside-down, will it right itself?			0.80
Yes (correct response)	21 (26)	8 (23)	
No (incorrect response)	61 (74)	27 (77)	
How long does it take for a sinking vehicle to become completely submerged?			0.45
2-4 minutes (correct response)	23 (28)	7 (20)	
Any other time value (incorrect response)	59 (72)	28 (80)	

^*^significant difference (*p* ≤ 0.05)

**Fig. 1:**
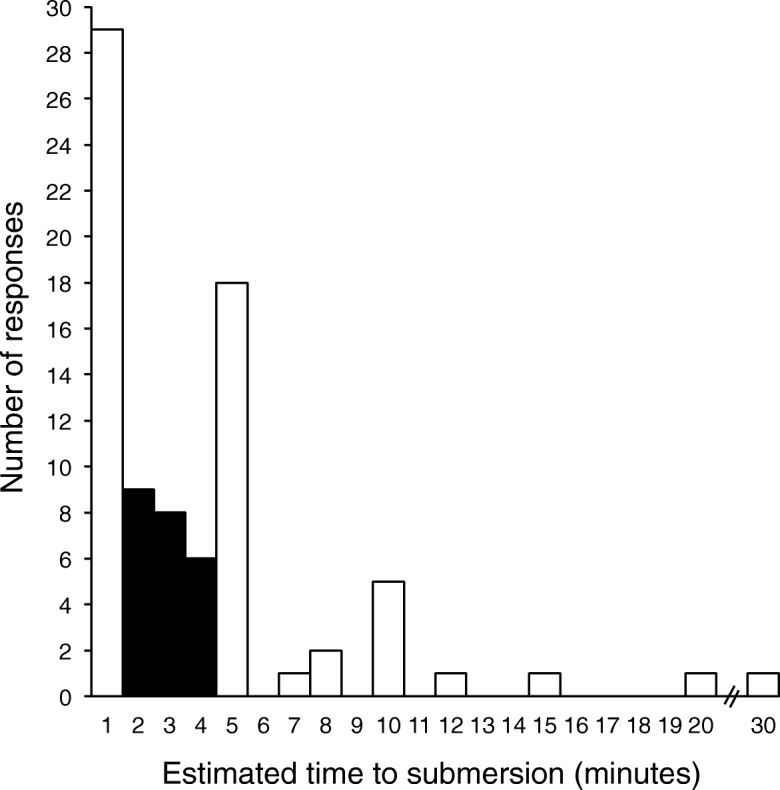
Respondent estimates for the time it would take for a vehicle to submerge below the surface of the water (*n* = 82)

Figure 1 shows the frequency of responses for estimated times for a sinking vehicle to completely submerge below the water surface. Only 23 (28%) respondents chose a correct value ranging from two to 4 min (Giesbrecht and McDonald 2010; Donohue 1991).

The final two “Knowledge” questions (open-ended) were completed for respondents who had “personal knowledge” of a vehicle submersion or were familiar with the “advised self-rescue protocol”. Ten respondents had personal knowledge of someone involved in a vehicle submersion incident, those who chose to provide additional details (*n* = 8) reported: 3 cases involved breaking through ice; 2 involved flooded/washed-out roads; 2 involved losing control of a vehicle and ending up in a body of water that was in close proximity (ditch and river); and 1 involved a heavy farm vehicle entering a water filled dug-out. The survival rate of the occupants in these incidents was approximately 67%.

The 5 respondents who were familiar with the advised self-rescue protocol were all from the “aware” group (all were “aware” from television).

### Public attitudes regarding vehicle submersion incidents

Responses to all “Attitude” based questions are presented in Table 3. The proportions of responses were not significantly different between the “aware” and “unaware” groups for any question.

**Table 3:** Responses to all “Attitude” based questions (*n* = 82). The final two questions were only for respondents who indicated they had a rescue or window-breaking tool in their vehicle (*n* = 13)

Questionnaire Item	Sample n (%)	Aware Group n (%)	Aware vs. Unaware *p*-value
Perceived risk of being involved (self) in a vehicle submersion			0.38
None	26 (32)	11 (31)	
Slight	45 (55)	19 (54)	
Moderate	8 (10)	5 (14)	
High	3 (4)	0 (0)	
Very high	0 (0)	0 (0)	
Perceived risk of others being involved in a vehicle submersion			0.24
None	16 (20)	8 (23)	
Slight	41 (50)	16 (46)	
Moderate	21 (26)	11 (31)	
High	4 (5)	0 (0)	
Very high	0 (0)	0 (0)	
Perceived chance of surviving a vehicle submersion			0.37
Slight	15 (18)	6 (17)	
Moderate	54 (66)	21 (60)	
High	13 (16)	8 (23)	
Perceived importance for “visible & reachable” rescue tool			0.82
Not important	0 (0)	0 (0)	
Slightly important	0 (0)	0 (0)	
Moderate importance	0 (0)	0 (0)	
High importance	5 (6)	2 (6)	
Very high importance	77 (94)	33 (94)	
Preferred colour of rescue tool			0.15
Black	10 (12)	5 (14)	
Green	10 (12)	6 (17)	
Orange	23 (28)	12 (34)	
Pink	13 (16)	2 (6)	
Red	14 (17)	7 (20)	
Yellow	12 (15)	3 (9)	
Rescue tool color to be most visible in the case of an emergency			0.65
Black	0 (0)	0	
Green	0 (0)	0	
Orange	67 (82)	28 (80)	
Pink	1 (1)	0 (0)	
Red	1 (1)	1 (3)	
Yellow	13 (16)	6 (17)	
Perceived confidence in finding rescue tool in an emergency			0.80
Not confident	0 (0)	0	
Reasonably confident	8 (62)	4 (57)	
Very confident	5 (38)	3 (43)	
Perceived confidence in using rescue tool in an emergency			0.80

### Public practice for vehicle submersions

Responses to “Practice” questions (6 close-ended, 1 open-ended), for which “correct” and “incorrect” response were assigned, are summarized in Table 4. Distributions of responses were not significantly different between the “aware” and “unaware” groups for any questions.

**Table 4:** Responses to “Practice” questions (*n* = 82; 6 closed-ended and 1 open-ended). All responses were categorized as “correct” or “incorrect”. For the open-ended question, responses were assigned as “correct” or “incorrect” window breaking objects/devices. Data for the final question was not available for all respondents (*n* = 71)

Questionnaire Item	Sample n (%)	Aware Group n (%)	Aware vs. Unaware *p*-value
First action selected if involved in a vehicle submersion			0.22
Seatbelt or window (*correct response*)	73 (89)	33 (94)	
Any other action (*incorrect response*)	9 (11)	2 (6)	
What would you use to break a car window			0.09
Rescue tool or heavy hard object (*correct response*)	47 (57)	24 (69)	
Body part or other item (*incorrect responses*)	35 (43)	11 (31)	
Best window to break			0.71
Any side window or sunroof (*correct response*)	60 (73)	25 (71)	
Front or rear windshield (*incorrect response*)	22 (27)	10 (32)	
Best place to strike a side window to break it			0.86
Lower corner closest to the hinges (*correct response*)	8 (10)	3 (9)	
Any other location (*incorrect response*)	74 (90)	32 (91)	
Window breaking device in the vehicle			0.46
Yes (*correct response*)	13 (16)	7 (20)	
No (*incorrect response*)	69 (84)	28 (80)	
Rescue tool location selected (initial response)			0.15
Rear view mirror (*correct response*)	11 (13)	7 (20)	
Any of the other locations (*incorrect response*)	71 (87)	28 (80)	
Rescue tool location that best suits “visible & reachable” criteria			0.89
Rear view mirror (*correct response*)	18 (25)	9 (26)	
Any of the other locations (*incorrect response*)	53 (75)	26 (74)	

**Fig. 2:**
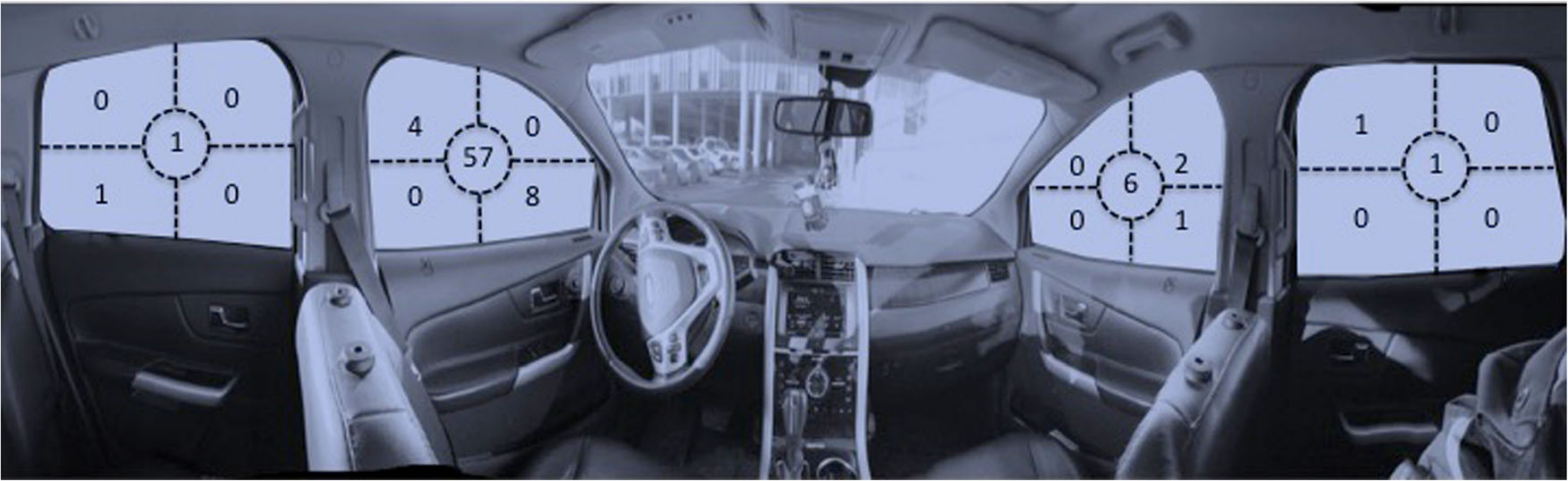
Panoramic view of vehicle side and front windows; front headrests were removed for clarity. Respondents selected which side window they would exit from, and then pointed to the specific location they would hit in order to break it (*n* = 82). Frequency is indicated within each window-area section (e.g., 4 corners and the center)

While sitting inside the demonstration vehicle, respondents were asked which side-window they would break, and the specific location they would hit to break that window (Fig. 2). Only 8 (10%) chose the best location (lower corner closest to the hinges, e.g., the front), while the majority (*n* = 65; 79.3%) chose to hit a window in the center area (Giesbrecht and McDonald 2010).

### Public practice for rescue tools

Thirteen (16%) respondents indicated they had some type of rescue tool in their vehicle (these included 7 center punches, 6 rescue hammers and 1 anti-theft device; one reported having 2 tools). Tool locations were also indicated (6, front center console; 3, glove box; 2, keychain; 1, driver side door compartment; 1, passenger side floor; and 1, back seat). No devices were hung from a rear-view mirror.

### Practice for child seat use

Of the 71 (87%) respondents who reported using a motor vehicle, 10 (14%) had 1 or more child seats. They were asked which way it faced, where it was located and what type of restraint/harness system was used. One had 1 rear-facing seat and six had 1 front-facing seat. Two respondents had 1 each of front- and rear-facing seats, while one had 2 front-facing seats. Twelve of the 13 seats were located in the back seat. Of the 3 rear-facing seats, 2 had chest harnesses while 1 used a seatbelt. Of the 10 front-facing seats, 7 had chest harnesses while 3 used seatbelts.

## Discussion

To our knowledge this is the first survey conducted to determine public knowledge, attitudes and practices regarding vehicle submersions. Respondents generally reflected the adult Canadian population and results provide some insights into present knowledge, and directions for future research and public education efforts. Most respondents were aware of media reports of vehicle submersions while 13% had personal knowledge of such incidents. The predominant attitude was that there was minimal risk of being involved in a vehicle submersion and that they would likely survive such an event. At the time of this survey, the effectiveness of *Operation ALIVE* educational initiatives was minimal; only 45% of respondents were aware of any of these initiatives, and only 14% of this “aware” group remembered hearing the specific escape protocol offered by this program.

Unexpectedly, most respondents selected a “successful” initial action that could lead to escape and survival during a vehicle submersion (e.g., either, “seatbelt off” or “window open”). Although either of those two actions could result in a successful exit it is not guaranteed. Respondents who chose to first open a side window may unfasten the seatbelt and exit successfully, however it is not guaranteed that respondents who first release a seatbelt will then open a side window to escape successfully (e.g., someone could release their seatbelt and then try to open a door, or make a cell phone for help, etc.). Minimally, survival depends on releasing a seatbelt and opening a window, and then exiting through that window as quickly as possible.

Additionally, most respondents chose a proper window to break and a proper device/object to break the window with. However, most do not carry such a device in their vehicle. Other responses to questions on knowledge (the poor understanding of the quick submersion time), attitude (the high perceived chance of survival) and practice (poor window selection, low tool availability and not knowing the best location to break window), generally indicated that the chances of an individual knowing and performing a complete self-rescue sequence, is unlikely.

One of the factors that emphasizes the need to know how to self-rescue, is the time sensitivity of this scenario and the current tendency to call emergency dispatch (e.g., 9–1-1) for help in all emergency situations. The chance of rescue by emergency response personnel is negligible, as occupants need to exit the vehicle within the first minute of immersion and rescue personnel will take longer to arrive on scene (McDonald and Giesbrecht 2013a; Giesbrecht and McDonald 2010). Although calling emergency dispatch is contraindicated, the International Academies of Emergency Dispatch developed new emergency dispatch protocols for “a vehicle in water” and “a vehicle in floodwater” scenarios in which dispatchers now instruct occupants on how to perform self-rescue according to the SWOC protocol (Giesbrecht 2016a; Giesbrecht 2016b).

### Relevance of results

It is very concerning that the majority of respondents do not know the “SWOC”, or similar, self-rescue protocol, or the short time available to perform the protocol (about 1 min). Therefore, it is very plausible that poor choices or incorrect decisions could waste time during a very stressful event and lead to unsuccessful escape. One of the authors previously described a model where high stress conditions, which allow less time for logical decision making, result in instinctive decisions and behaviour (Giesbrecht 2005). These decisions often result in unsafe or erroneous actions (Reason 1992) due to factors such as relying on incorrect information; rejecting or misapplying correct information; or choosing inappropriate actions (including doing nothing). Education was proposed as one of the main factors that could reduce errors and unsafe actions (Giesbrecht 2005). Seventy-two percent of respondents had poor knowledge of submersion time, those with a shorter estimation may be more likely to panic because they don’t think they have enough time to make good decisions and actions. Alternatively, those with longer estimations may believe they have lots of time to do things like make a phone call for help; these actions will likely waste the first minute during which escape is possible.

Another factor that reduces the chance of survival is that escape is not a one-step process and each incorrect choice will increase escape time and reduce the success rate. For example, one quarter of respondents chose to break front or back windshields, which are virtually impossible to break apart because they are laminated and/or impractical to hit effectively (especially the back windshield). Most chose to hit the side window in the center to break it, however, this area is least rigid, absorbs more energy, and is less effective than the most rigid area near the doorframe closest to the hinges. While few respondents actually have commercial window breaking tools, none have them in locations that best meet appropriate criteria of being “visible and reachable” when needed. Thus, the probability of these tools being accessed in a stressful vehicle submersion is also lower.

Although *Operation ALIVE* advocates hanging rescue tools from the rear-view mirror, many respondents in the test vehicle initially chose a tool from a sun visor or key chain location. Although the visor is potentially a good location, when it is lowered, the tool is obscured and not visible. Similarly, a key chain is a seemingly good location for a rescue tool, but is not recommended for several reasons including: low probability of being noticed when the occupant is under stress; many positions of the steering wheel block vision of the key chain; the tool is not guaranteed to be in the vehicle if all drivers of that vehicle don’t also have a tool on their key chains; and the trend towards keyless ignitions will increase the probability that key FOBs remain in pockets, hand bags, etc. and are therefore not visible.

Generally, both hand-crank and electric windows work, and can be opened, when a vehicle first enters the water, assuming no major structural damage. If windows are not functional, having a tool that is both “visible and reachable” is highly valuable.

There is one other significant problem with reliance on breaking windows. As of September 1, 2017, most vehicles in the North America are manufactured with laminated glass in side windows for ejection mitigation, making them virtually impossible to break. Thus, it is important for the public to know, remember, and follow the simple advice to quickly undo seatbelts, open the windows and exit the vehicle. Also, since electric windows should work if activated quickly (Buning et al. 2008), the need to break a window is generally avoidable.

### Implications for future work

Public knowledge, attitudes and practices related to vehicle submersion are poor. Simple education of the proper self-rescue protocols has the potential to help lower this high fatality rate. This study indicates that awareness of the *Operation ALIVE* program (43%) did not, at this point, increase general knowledge or quality of choices made by respondents. This indicates that we need to embark on more effective strategies at the national and international levels. More work should also be considered to specifically target middle- and low-income countries.

Given the importance of prior knowledge and education in improving the probability of an individual making a correct decision(s) under stress (Giesbrecht 2005; Leach 1994; Reason 1992), it is clear that public education efforts should be increased and improved. One obvious strategy is to standardize an educational program for schools, much like fire safety programs. Topics should include the standard advice “Seatbelts, Windows, Out, Children first (SWOC)”, but also include advice regarding the order in which children should be released (from the oldest to youngest), the best rescue tool type and location for installation and finally, proper actions after successfully exiting a sinking vehicle.

In conjunction with the development of knowledge translation strategies, corresponding multi-center research could be conducted to determine message penetration, retention, acceptance and compliance. Further KAP surveys could also be conducted with larger samples from multiple locations (importantly including low- and middle-income nations) and increase the scope of questions such as: a complete list of self-rescue actions; the proper order of children to be assisted out of the vehicle etc.

Finally, prevention efforts should continue on road signage, design, barriers, flood warning systems, etc. (Wintemute et al. 1990). It would also be beneficial to introduce an automatic window opening device (Giesbrecht et al. 2017) that could provide a guaranteed exit and remind occupants to exit through the windows, and eliminate the limitation of unbreakable laminated side windows.

### Summary

Although most respondents were aware of vehicle submersion incidents through the media, they had a poor understanding of vehicle submersion incidents. This situation should be rectified because even though the probability of being in a vehicle submersion is low, this type of accident has one of the highest mortality rates of any type of single-vehicle accident, and death by drowning is usually preventable. Specifically, education should focus on the following points: passengers have only about 1 min to self-exit from a sinking vehicle; they should not rely on calling emergency dispatch; rather they should follow the SWOC protocol [SEATBELTS off, WINDOWS open or broken, OUT immediately, CHILDREN first (youngest to oldest)]; and safety devices such as a window-breaking center punch should be small and hung from the rear-view mirror. Finally, more work is required for future knowledge translation, public research and education initiatives to prevent vehicle submersion deaths, with consideration for different strategies in low-, middle- or high-income nations.
